# Nonsurgical treatment outcomes for surgical candidates with lumbar disc herniation: a comprehensive cohort study

**DOI:** 10.1038/s41598-021-83471-y

**Published:** 2021-02-16

**Authors:** Chi Heon Kim, Yunhee Choi, Chun Kee Chung, Ki-Jeong Kim, Dong Ah Shin, Youn-Kwan Park, Woo-Keun Kwon, Seung Heon Yang, Chang Hyun Lee, Sung Bae Park, Eun Sang Kim, Hyunsook Hong, Yongeun Cho

**Affiliations:** 1 Department of Neurosurgery, Seoul National University College of Medicine, 103 Daehak-ro, Jongno-gu, Seoul, 03080 South Korea; 2 Department of Neurosurgery, Seoul National University Hospital, 101 Daehak-ro, Jongno-gu, Seoul, 03080 South Korea; 3 Division of Medical Statistics, Medical Research Collaborating Center, Seoul National University Hospital, 101, Daehak-ro, Jongno-gu, Seoul, 03080 South Korea; 4 Department of Brain and Cognitive Sciences, Seoul National University, 1 Gwanak-ro, Gwanak-gu, Seoul, 08826 South Korea; 5 Department of Neurosurgery, Seoul National University Bundang Hospital, 82 Gumi-ro 173 Beon-gil, Bundang-gu, Seongnam-si, Gyeonggi-do 13620 South Korea; 6 Department of Neurosurgery, Yonsei University Health System, 50-1 Yeonsei-ro, Seodaemun-gu, Seoul, 03722 South Korea; 7 Department of Neurosurgery, Yonsei University College of Medicine, 50-1 Yeonsei-ro, Seodaemun-gu, Seoul, 03722 South Korea; 8 Department of Neurosurgery, Korea University Guro Hospital, 148 Gurodong-ro, Guro-gu, Seoul, 08308 South Korea; 9 Department of Neurosurgery, Korea University College of Medicine, Korea-daero 73, Seongbuk-gu, Seoul, 02841 South Korea; 10 Department of Neurosurgery, Seoul National University Boramae Hospital, Borame Medical Center 20, Boramae-ro 5-gil, Dongjak-gu, Seoul, 07061 South Korea; 11 Department of Neurosurgery, Spine Center, School of Medicine, Samsung Medical Center Sungkyunkwan University, Seoul, Korea; 12 Department of Neurosurgery, Gangnam Severance Hospital, 211 Eonju-ro, Gangnam-gu, Seoul, 06273 South Korea

**Keywords:** Pain, Musculoskeletal system, Pain management

## Abstract

Physicians often encounter surgical candidates with lumbar disc herniation (LDH) who request non-surgical management even though surgery is recommended. However, second opinions may differ among doctors. Therefore, a prospective comprehensive cohort study (CCS) was designed to assess outcomes of nonsurgical treatment for surgical candidates who were recommended to undergo surgery for LDH but requested a second opinion. The CCS includes both randomized and observational cohorts, comprising a nonsurgery cohort and surgery cohort, in a parallel fashion. Crossover between the nonsurgery and surgery cohorts was allowed at any time. The present study was an as-treated interim analysis of 128 cases (nonsurgery cohort, n = 71; surgery cohort, n = 57). Patient-reported outcomes included visual analogue scores for the back (VAS-B) and leg (VAS-L), the Oswestry Disability Index, the EuroQol 5-Dimension instrument, and the 36-Item Short-Form Health Survey (SF-36), which were evaluated at baseline and at 1, 3, 6, 12, and 24 months. At baseline, age and SF-36 physical function were significantly lower in the surgery cohort than in the nonsurgery cohort (p < 0.05). All adjusted outcomes significantly improved after both nonsurgical and surgical treatment (p < 0.05). The nonsurgery cohort showed less improvement of VAS-B and VAS-L scores at 1 month (p < 0.01), but no difference between cohorts was observed thereafter for 24 months (p > 0.01). Nonsurgical management may be a negotiable option even for surgical candidates in the shared decision-making process.

## Introduction

In recent years, the number of lumbar spinal surgeries has been increasing, leading to increased use of medical resources, including both surgery and nonsurgical treatments such as exercise, medication, physiotherapy, and other interventions^1,2^. Lumbar disc herniation (LDH) accounts for approximately two-thirds of spinal pain diagnoses, and many studies have examined the optimal utilization of medical resources^3–5^. Many prospective studies have compared the effectiveness of surgery versus nonsurgical interventions. Although surgery has shown better outcomes in the short- or mid-term^6–9^, the effect of surgery does not always last over the long term.^9,42,43^ A systematic review of accumulated evidence led to the conclusion that surgery resulted in faster relief of symptoms, but the ultimate long-term outcomes were similar between nonsurgery and surgery groups^10^. Usually, surgery is recommended when nonsurgical treatments fail to relieve symptoms of LDH^11–14^. However, not all patients with current surgical indications want to receive surgery. Physicians often encounter surgical candidates who request nonsurgical management even though surgery is recommended. Their reasons include fear of surgery, hopes for spontaneous improvement, and a lack of regard for the modest benefits of surgery, especially in the long term^12,15^. However, the outcomes of surgical versus non-surgical treatments were not clear in this specific setting. Therefore, we designed a prospective study to assess the outcomes of nonsurgical treatment for surgical candidates who opted for nonsurgical management. In clinical research, randomized trials are widely accepted as the definitive method of evaluating the efficacy of therapies^16^. However, in real-world clinical research, many patients do not consent to randomization^16^. Therefore, a comprehensive cohort study (CCS) was designed to respect the preferences of all patients fulfilling the clinical eligibility criteria regardless of their consent to undergo randomization. Thus, the CCS included both randomized and observational cohorts of subjects who consented to participate in the study but declined to undergo randomization^16^. The result of this study may reveal outcomes in a real-world situation. The study was planned to compare outcomes for at least 2 years of follow-up of enrolled patients, but an interim analysis was planned 2 years after initiating the study to prevent patients from being exposed to unreasonable risks and to avoid imposing the burden of a clinical trial without having a reasonable expectation that the trial would produce useful information^17^. The aim of this article is to provide the results of the interim analysis.

## Methods

### Patients and design of study (details in supplement 1)

**Figure 1:**
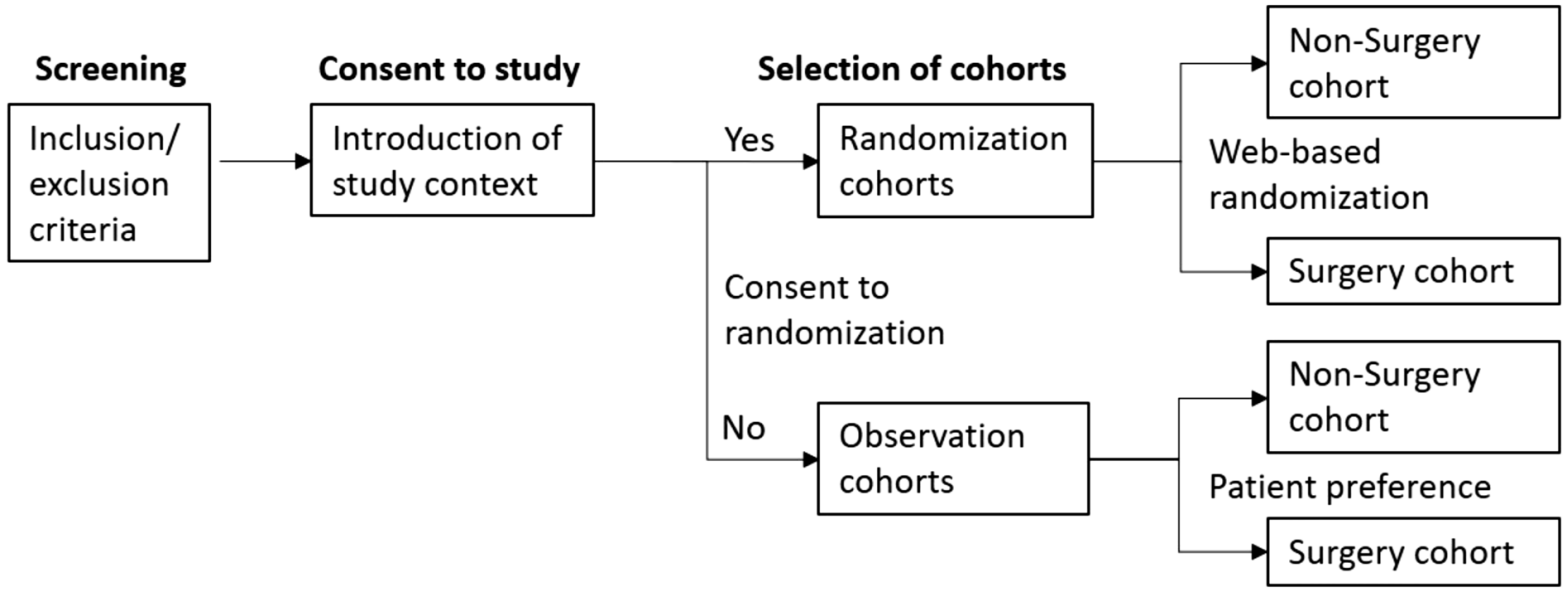
Comprehensive cohort study design. An enrollment of participants consists of three steps: screening, consent for study and selection of cohorts. A web-based system was used across participating hospitals for randomization.

The present study intended to compare nonsurgical and surgical outcomes of lumbar disc herniation (LDH) in patients who voluntarily visited a clinic for a second opinion after surgery was recommended by another physician who actively treats spinal disease (spinal physicians). The enrollment process consisted of three steps (Table 1): screening according to inclusion/exclusion criteria (Table 1), consent for inclusion in the study and selection of inclusion in the randomization or observational cohorts (Fig. 1). Attending surgeons and research coordinators (registered nurses) participated in all steps. Participants were allowed to cross over to the other treatment cohort or to withdraw from participation at any time. A web-based system was used across participating hospitals for randomization and data registration. The follow-up schedules were the same for both study cohorts and regular patients, and their outcomes were evaluated during clinic visits or via telephone at 1, 3, 6, and 12 months after the initiation of treatment and yearly thereafter. The present study did not provide any reward to patients, and their schedule in the clinic was the same as that of regular patients. The research team tried to ensure study subjects’ follow-up rate by managing clinic schedules and contacting the subjects via telephone while respecting their voluntary participation. As such, any possible negative effects of the study were minimized. The independent data safety monitoring board (DSMB) reviewed the study every 6 months. This study was approved by the institutional ethical review board of each university hospital (H 1605-013-759, 4-2106-0492, and B1603/337-004) and registered at both clinicaltrials.gov (NCT02883569, first posted on Aug/30/2016) and the Clinical Research Information Service (https://cris.nih.go.kr/cris/en/) (KCT0000203). All research was performed in accordance with relevant laws/guidelines/regulations of the Republic of Korea, and the present study was conducted in accordance with the principles of the Declaration of Helsinki. Written informed consent was obtained from all participants and/or their legal guardians.

**Table 1:** Inclusion and Exclusion criteria.

Inclusion criteria	≥ 18 years of age Symptomatic single-level lumbar disc herniation (LDH) (protrusion, extrusion, or sequestration) documented by magnetic resonance imaging (MRI) taken within 3 months from the clinic visit date Intractable leg pain of more than 5/10 on a visual analogue scale (VAS) for at least 6 weeks despite active non-surgical treatments such as exercise, physiotherapy, medications, or epidural injections Concordant neurological symptoms and signs such as numbness, radiating pain, mild weakness (stronger than manual motor power grade IV/V), and/or limitations on the straight leg raise test (< 60°) Voluntarily agree to study participation and sign a written informed consent form Fully understand the details of a clinical study and those who are cooperative
Exclusion criteria	Combined significant weakness (motor power grade ≤ 3/5) or cauda equina syndrome, segmental instability (angular motion ≥ 15° and/or translation ≥ 4 mm) Trauma-associated LDH Combined neurological disease (such as Parkinson disease), inflammatory joint disease, tumor, infection, and neuropathy Previous history of spinal surgery Pregnancy

### Study intervention

#### Nonsurgical treatment

Because all participants were surgical candidates, surgery would have been the natural course of events. Therefore, in the present study, the intervention was nonsurgical treatment, performed by pain physicians and rehabilitation physicians. Every non-surgical treatment was entrusted to pain physicians and rehabilitation physicians, who were blinded to the study. The treatment was not uniform among all patients and was customized based on the patients’ individual symptom/signs. However, all treatment decisions followed the same principles. After a review of previous treatments, a combination of noninvasive interventions such as lifestyle modifications, exercise, physiotherapy, and/or medication (nonsteroidal anti-inflammatory drugs and/or weak opioids) was applied for several weeks to relieve patients’ symptoms^1,2^. If these noninvasive treatments were not effective, other nonsurgical treatments, such as manual manipulation, therapeutic massage, and injection-based treatments, were performed for another several weeks^1,2^. If these treatments were not effective, percutaneous adhesiolysis was recommended^2^. The patients had the right to request surgery if those treatment were not effective at any time.

### Surgical treatment

All surgeons had more than 5 years of experience with either standard open microscopic discectomy or full endoscopic lumbar discectomy^11,18–21^. The detailed standard surgical procedures were shared by all surgeons and researchers, and each surgeon was asked to operate using the routine surgical technique with which he or she was most confident. All operations were performed with patients in the prone position under general anesthesia. Standard microscopic discectomy was performed after midline or paraspinal skin incision. After partial hemilaminectomy, the herniated disc material was identified using a surgical microscope, and sufficient decompression was confirmed by free mobility of the affected nerve root. Full endoscopic procedures were performed as previously described^19,22–24^. Two approaches were used according to the level of LDH. Generally, a transforaminal approach was used for LDH located at L4-5 or above, and an interlaminar approach was used for LDH at L5-S1. After surgery, all patients were encouraged to ambulate from the day of surgery, and they were discharged at postoperative day 1 or 2. No lumbar supporting braces were applied, but strenuous activities such as sports, certain leisure activities or weightlifting were not allowed until 3 months after surgery. Patients were scheduled to visit the clinic at postoperative months 1, 3, 6, and 12, and yearly thereafter.

### Outcome measurements

All participants were asked to complete patient-reported outcome (PRO) questionnaires that contained VAS scores for the back (VAS-B, x/10) and leg (VAS-L, x/10), the Korean version of the Oswestry Disability Index (K-ODI, x/45)^25^, quality of life measurements from the EuroQol 5-Dimension instrument (EQ-5D, https://euroqol.org/eq-5d-instruments/eq-5d-5l-about/), and the 36-Item Short-Form Health Survey (SF-36) at every clinic visit. Responses to the EQ-5D descriptive system were normalized to a range from “health worse than death,” represented by a score of − 1, to “perfect health,” represented by a score of 1^26^. The EQ visual analogue scale (EQ-VAS), which describes perceptions of health, was scaled from 0 (worst health) to 100 (best health). The SF-36 consists of eight sections, including vitality (VT), physical functioning (PF), bodily pain (BP), general health perceptions (GH), physical role functioning (RP), emotional role functioning (RE), social role functioning (SF), and mental health (MH). Each section was transformed to a score from 0 to 100, with higher scores meaning less disability and lower scores meaning greater disability^6,27,28^.

### Statistical analysis (details in supplement 2)

An interim analysis was planned to meet the requirements of governmental funding in the 2nd year and to decide whether to extend the study for a longer follow-up period. Because the proportions of cross-over between surgery cohort and non-surgery cohort were expected to be high considering previous SPORT trial^11^, the outcomes of the actually received treatments were analyzed (as-treated analysis), with comparison between as-treated (actual treatments received) surgery cohort and the non-surgery cohort. The outcomes of the interim analysis were the changes in VAS-B, VAS-L, K-ODI, EQ-5D, EQ-VAS, and each section of SF-36 from the baseline measures during the follow-up period. A generalized linear mixed-effect model was utilized to compare clinical outcomes between the surgery and nonsurgery cohorts and to address a patient-specific trend in the outcomes. Adjusting the cofounding variables, the adjusted mean difference between the surgery and nonsurgery cohorts was estimated based on the mixed models. When group comparisons were performed at each measurement time due to significant interaction between the cohort and measurement time, the adjusted p-value and 99% confidence interval were estimated by the Bonferroni method to control type I error inflation due to multiple testing. Based on the hazard ratio of significant factors characterizing the surgery cohort, a formula for the surgery preference score was produced. The optimal cutoff value of the surgery preference score to discriminate the surgery cohort from the nonsurgery cohort was determined using the minimum p-value approach and validated using twofold cross validation^29,30^. All statistical analyses were performed using SAS version 9.4 (SAS Institute, Cary, NC, USA), and statistical significance was defined as p < 0.05 (two-sided).

## Results

### Enrollment

**Figure 2:**
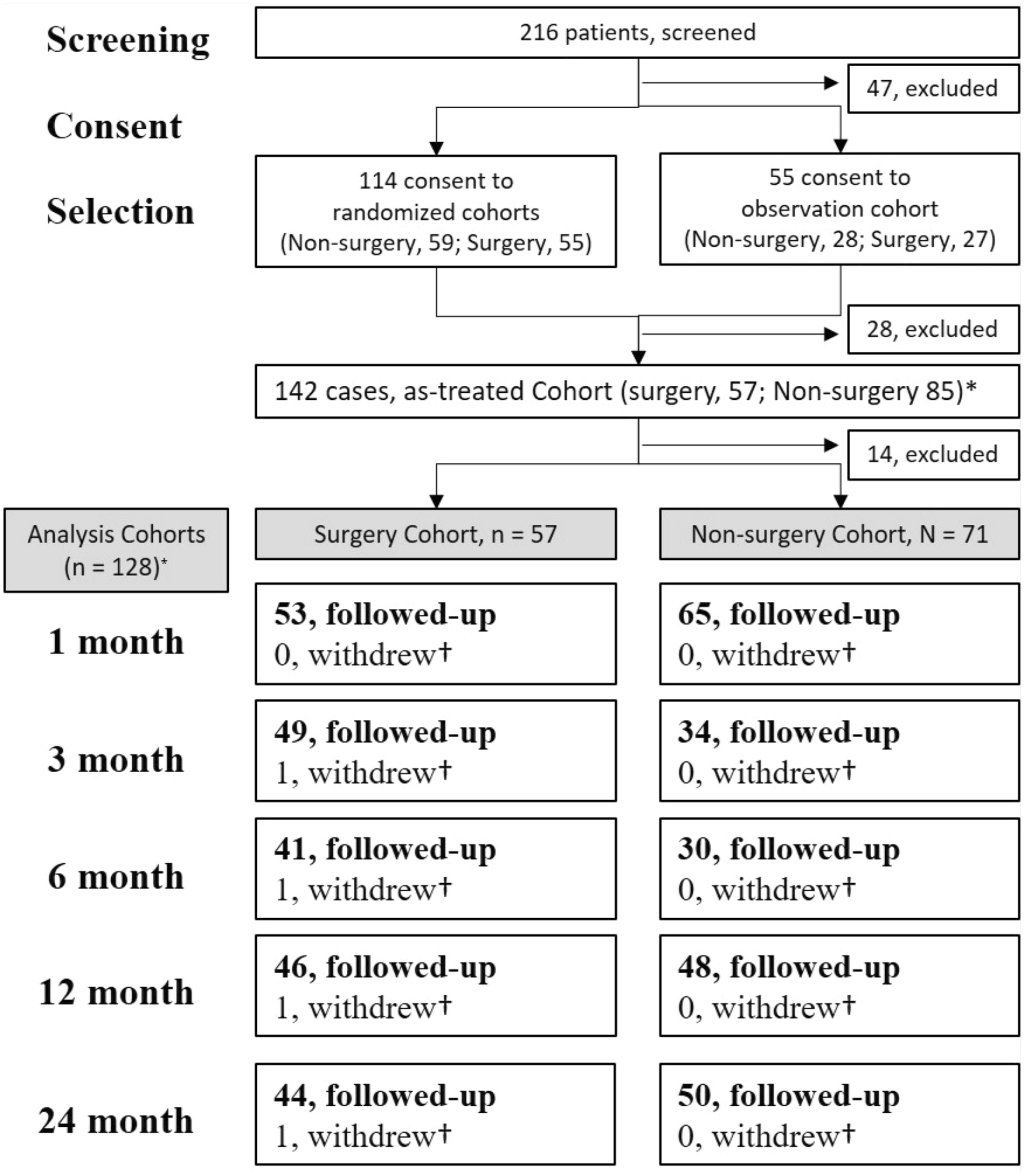
Flow chart of patients. Overall, 128 cases (nonsurgery, n = 71 [55%]; surgery, n = 57 [45%]) were included in the present analysis cohorts. Initially, 216 patients were screened, 169 patients consented to participate. Randomized cohort was selected by 114 patients and observational cohort was selected by 55 patients. 142 cases were included in the as-treated cohorts, but 14 patients missed visit. The number of patients with registered data are described in the boxes. *One patient was included in both the nonsurgery and surgery cohorts in the as-treated analysis, because the participant allocated in the surgery cohort received nonsurgical treatment for 9 months before undergoing surgery 9 months after enrollment. ^†^Cumulative over time.

Overall, 128 cases (nonsurgery, n = 71 [55%]; surgery, n = 57 [45%]) were included in the present analysis cohorts (Fig. 2). During the enrollment period, 216 patients were screened, and 169 patients consented to participate in the study and selected cohorts (Fig. 2). Of 141 participants, 114 patients selected the randomized cohort and were allocated to either the nonsurgery cohort (n = 59) or the surgery cohort (n = 55). The other 55 patients selected the observational cohort and chose nonsurgery (n = 28) or surgery (n = 27). After selection, 28 patients did not visit the clinic again and were excluded from the study (Fig. 2). After the initiation of treatment, 14 patients withdrew or did not visit the clinic and were excluded from the analysis (Fig. 2). In the randomized cohorts, 27% (16/59) of participants in the nonsurgery cohort and 51% (28/55) of participants in the surgery cohort crossed over to the other treatment cohort during follow-up. One patient in the randomized cohort who was allocated to the surgery cohort received nonsurgical treatment for 9 months and underwent surgery 9 months after enrollment. This individual was included in both the nonsurgery and surgery cohorts in the as-treated analysis. Not all patients attended every scheduled clinic visit or could be reached via telephone, and the number of available data points was not the same at each time point (Fig. 2). At 12 months, 48/71 (67.6%) patients in the nonsurgery cohort and 46/57 (80.7%) patients in the surgery cohort were followed-up. The data for 50/71 (70.4%) patients in the nonsurgery cohort and 44/57 (77.2%) patients in the surgery cohort were available for analysis at 24 months (Fig. 2).

### Characteristics of cohorts

The characteristics of participants in both cohorts are summarized in Table 2. The mean age of participants in the surgery cohort (46.0 years) was lower than that of participants in the nonsurgery cohort (50.8 years) (p = 0.04). The most common level was L4-5 (51% in the nonsurgery cohort and 62% in the surgery cohort), followed by L5-S1 (38% in the nonsurgery cohort and 35% in the surgery cohort). Occupational activity (OA) was classified into three categories: high OA, intermediate OA, and low OA^31^. Patients in the surgery cohort reported greater leg pain (p = 0.02) and lower scores on function or quality of life-related questionnaires than those in the nonsurgery cohort, such as the K-ODI (p < 0.01), EQ-5D (p < 0.01), and 4 sections of the SF-36 (PF, MH, SF, and BP) (p < 0.05).

**Table 2:** Characteristics of cohorts.

	Surgery (n = 57)	Non-surgery (n = 71)	p-value
Age, mean ± SD	46.02 ± 13.82	50.77 ± 12.98	0.04*
Female, n (%)	26 (45.6%)	38 (53.5%)	0.38††
Weight	71.55 ± 12.98	66.67 ± 11.60	0.03†
Height	168.59 ± 7.97	164.78 ± 9.07	0.02†
BMI (kg/m^2^)	25.06 ± 3.50	164.78 ± 9.07	0.30†
Levels of herniation			0.50**
L1–2	0 (0.0%)	1 (1.4%)	
L2–3	0 (0.0%)	1 (1.4%)	
L3–4	2 (3.5%)	6 (8.5%)	
L4–5	35 (61.4%)	36 (50.7%)	
L5–S1	20 (35.1%)	27 (38.0%)	
Type of herniation			0.29††
Protrusion	15 (26.3%)	28 (39.4%)	
Extrusion	27 (47.4%)	28 (39.4%)	
Sequestration	15 (26.3%)	15 (21.1%)	
Occupational activity			0.14††
High	8 (14.0%)	3 (4.2%)	
Intermediate	35 (61.4%)	50 (70.4%)	
Low	14 (24.6%)	18 (25.4%)	
Diabetes, n (%)	2 (3.5%)	4 (5.6%)	0.69**
Hypertension, n (%)	7 (12.3%)	14 (19.7%)	0.26††
Smoking, n (%)	12 (21.1%)	7 (9.9%)	0.08††
VAS-B	5.67 ± 2.88	6.10 ± 2.13	0.67*
VAS-L	7.05 ± 2.21	6.27 ± 2.06	0.02*
K-ODI	23.53 ± 7.51	18.38 ± 7.22	< 0.001†
EQ-5D utility score	0.43 ± 0.19	0.57 ± 0.17	< 0.001*
EQ-VAS	49.02 ± 18.14	55.28 ± 16.56	0.05*
SF-36			
Physical functioning	30.35 ± 23.75	47.25 ± 25.87	< 0.001*
Physical role functioning	15.35 ± 27.85	22.89 ± 32.39	0.14*
Emotional role functioning	38.01 ± 36.43	48.36 ± 40.15	0.13*
Vitality	36.05 ± 16.22	40.14 ± 16.99	0.10*
Mental health	49.54 ± 18.19	56.85 ± 18.13	0.01*
Social role functioning	35.75 ± 20.24	47.18 ± 22.97	0.01*
Bodily pain	20.44 ± 17.15	33.52 ± 16.70	< 0.001*
General health perception	53.07 ± 18.99	50.92 ± 17.47	0.42*
Surgical treatment			
Open discectomy	23 (40.1%)	0 (0.0%)	
Endoscopic discectomy	34 (59.6%)	0 (0.0%)	
Non-surgical treatment			
Exercise only	0 (0.0%)	8 (11.3%)	
Medication only	0 (0.0%)	36 (50.7%)	
Intervention only	0 (0.0%)	3 (4.2%)	
Exercise + medication	0 (0.0%)	7 (9.9%)	
Medication + intervention	0 (0.0%)	15 (21.1%)	
Exercise + medication + intervention	0 (0.0%)	2 (2.8%)	

*BMI* body mass index, *VAS-B* visual analogue scale for back pain, *VAS-L* visual analogue scale for leg pain, *K-ODI* Korean version of the Oswestry Disability Index, *EQ-5D score* EuroQol 5-Dimension instrument score, *EQ-VAS* EuroQol visual analogue scale, *SF-36* 36-Item Short-Form Health Survey.

*Wilcoxon rank-sum test.

^†^t-test.

^††^Chi-square test.

**Fisher exact test.

### Clinical outcomes

**Figure 3:**
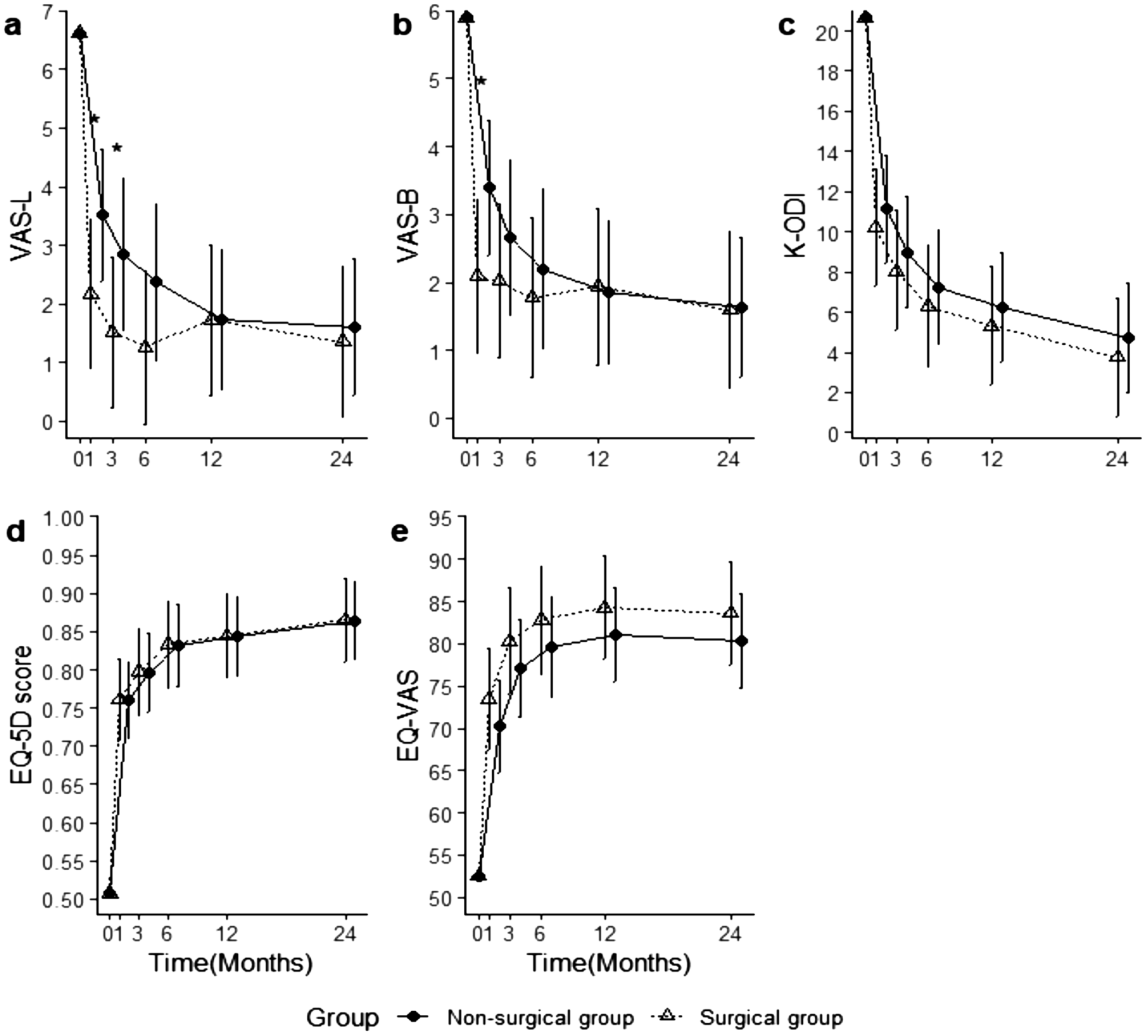
Clinical outcomes. The adjusted means and confidence intervals estimated from mixed effect models for the clinical outcomes; (**a**) Visual analogue scale for leg pain (VAS-L), (**b**) Visual analogue scale for back pain (VAS-B), (**c**) Korean version of the Oswestry Disability Index (K-ODI), (**d**) The EuroQol 5-Dimension utility score (EQ-5D score), (**e**) The EuroQol visual analogue scale (EQ-5D VAS). Asterisks (*) indicate significant differences between cohorts.

**Figure 4:**
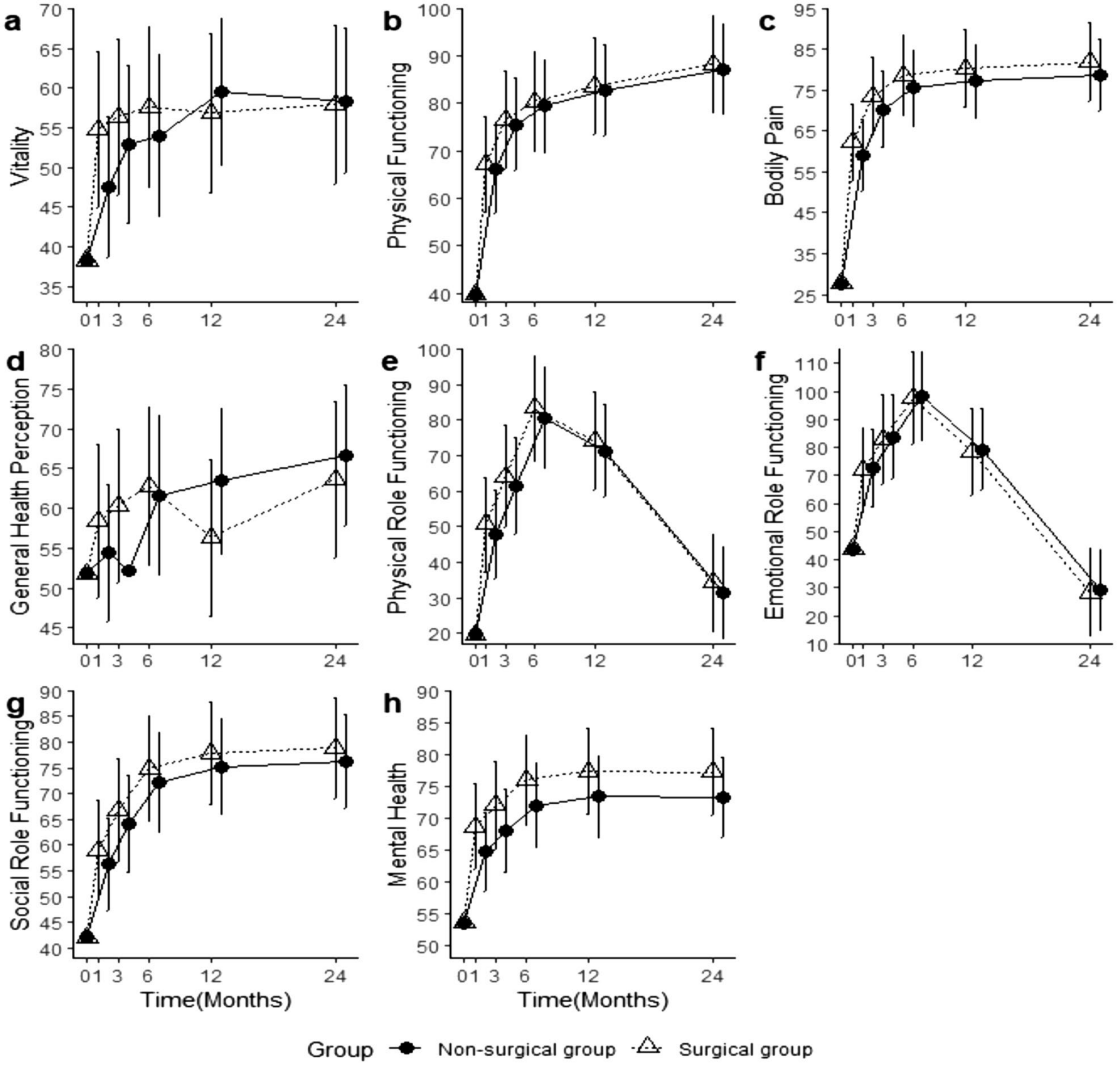
36-Item Short-Form Health Survey (SF-36) outcomes. The adjusted means and confidence intervals estimated from mixed effect models for 36-Item Short-Form Health Survey (SF-36) outcomes; (**a**) Vitality, (**b**) Physical Functioning, (**c**) Bodily Pain, (**d**) General Health Perceptions, (**e**) Physical Role Functioning, (**f**) Emotional Role Functioning, (**g**) Social Role Functioning, (**h**) Mental Health.

The mean $\pm$ standard deviation corresponding to each cohort at each time point and adjusted mean difference between cohorts are presented in Table 3. The adjusted mean and confidence interval for each cohort at each time point are shown in Figs. 3 and 4. Preoperative VAS-L scores were lower in the nonsurgery cohort than in the surgery cohort (p = 0.02). The VAS-L score improved significantly more in the surgery cohort than in the nonsurgery at 1 month, (p = 0.01) (Table 3 and Fig. 3a). After significant improvement of VAS-L at 1 month, it remained stationary in the surgery cohort for 24 months (p = 0.12), while it decreased further in the nonsurgery cohort for 24 months (p < 0.001). Eventually, VAS-L was not significantly different between the cohorts at 3 months after treatment and 24 months post-treatment (p > 0.01) (Table 3 and Fig. 3a). Similarly, VAS-B decreased significantly more in the surgery cohort than in the nonsurgery cohort at 1 month (p < 0.01), but the difference between cohorts was not observed thereafter during 24 months (p > 0.01) (Table 3 and Fig. 3b). The preoperative K-ODI, EQ-5D utility score, and EQ-VAS scores were better in the nonsurgery cohort than in the surgery cohort (p < 0.05) (Table 2). Although those parameters were different at baseline, those parameters were improved in both cohorts (p < 0.01) without difference between cohorts (p > 0.01) during 24 months (Table 3 and Fig. 3c–e). The SF-36 section parameters significantly improved in both the nonsurgery and surgery cohorts throughout the 24-months following treatment (p < 0.05) (Table 3 and Fig. 4), except for SF-36 (VT). SF-36 (VT) was not significantly different between cohorts during 24 months, but the change within each cohort was different. VT improved in both cohorts at 1 month; it did not further improve in the surgery cohort (p = 1.00) but did further improve in the nonsurgery cohort (p < 0.001) (Table 3 and Fig. 4a).

**Table 3:** Clinical outcomes.

	Pre-operation		1 month		3 months		6 months		12 months		24 months	p-value^‡^
**K-ODI**												
Surgery	23.53 ± 7.51		13.06 ± 7.81		10.04 ± 5.81		7.90 ± 5.66		8.07 ± 6.33		6.73 ± 6.99	< .001
Non-surgery	18.38 ± 7.22		12.20 ± 7.11		12.06 ± 6.75		11.07 ± 5.29		7.19 ± 5.19		6.00 ± 4.64	
Adjusted mean difference [95% CI]* (p-value^†^)	− 0.94 [− 3.00, 1.12] (0.37)											
**VAS-B**												
Surgery	5.67 ± 2.88		2.43 ± 1.88		2.33 ± 1.56		2.02 ± 1.37		2.24 ± 1.73		2.00 ± 1.73	0.86
Non-surgery	6.10 ± 2.13		3.97 ± 2.17		3.38 ± 2.32		3.10 ± 2.01		2.42 ± 1.67		2.35 ± 1.81	< .001
Adjusted mean difference [99% CI] (p-value)			− 1.31 [− 2.23, − 0.38] (0.002)		− 0.63 [− 1.66, 0.40] (0.57)		− 0.43 [− 1.52, 0.67] (1.00)		0.08 [− 0.92, 1.07] (1.00)		− 0.04 [− 1.03, 0.95] (1.00)	
**VAS-L**												
Surgery	7.05 ± 2.21		2.75 ± 2.34		2.12 ± 2.01		1.80 ± 1.65		2.28 ± 2.12		1.98 ± 1.80	0.12
Non-surgery	6.27 ± 2.06		3.97 ± 2.24		3.68 ± 2.31		3.20 ± 2.23		2.15 ± 1.97		2.16 ± 2.04	< .001
Adjusted mean difference [99% CI] (p-value)			− 1.34 [− 2.41, − 0.28] (0.01)		− 1.32 [− 2.51, − 0.14] (0.02)		− 1.12 [− 2.38, 0.14] (0.11)		0 [− 1.14, 1.14] (1.00)		− 0.25 [− 1.39, 0.89] (1.00)	
**EQ-5D utility score**												
Surgery	0.43 ± 0.19		0.73 ± 0.15		0.78 ± 0.08		0.81 ± 0.09		0.81 ± 0.13		0.84 ± 0.12	< .001
Non-surgery	0.57 ± 0.17		0.73 ± 0.13		0.75 ± 0.15		0.79 ± 0.08		0.82 ± 0.10		0.83 ± 0.10	
Adjusted mean difference [95% CI] (p-value)	0.00 [− 0.04, 0.04] (0.96)											
**EQ-5D VAS**												
Surgery	49.02 ± 18.14		71.23 ± 18.45		77.76 ± 15.04		79.95 ± 13.38		79.91 ± 9.30		80.45 ± 11.60	< .001
Non-surgery	55.28 ± 16.56		67.03 ± 17.87		73.09 ± 15.76		75.83 ± 10.09		80.58 ± 9.90		77.55 ± 11.72	
Adjusted mean difference [95% CI] (p-value)	3.23 [− 0.73, 7.2] (0.11)											
**SF-36 (VT)**												
Surgery	36.05 ± 16.22		52.74 ± 16.54		54.59 ± 16.42		55.61 ± 15.42		53.48 ± 17.32		56.70 ± 17.45	1.00
Non-surgery	40.14 ± 16.99		47.77 ± 17.34		50.15 ± 19.67		49.67 ± 21.85		58.13 ± 16.75		57.55 ± 14.23	< .001
Adjusted mean difference [99% CI] (p-value)			7.25 [− 0.73, 15.23] (0.10)		3.43 [− 5.44, 12.31] (1.00)		3.56 [− 5.81, 12.93] (1.00)		− 2.72 [− 11.25, 5.82] (1.00)		− 0.45 [− 8.94, 8.05] (1.00)	
**SF-36 (PF)**^**††**^												
Surgery	30.35 ± 23.75		58.30 ± 27.03		71.33 ± 22.19		75.37 ± 21.08		75.87 ± 23.05		80.80 ± 22.87	< .001
Non-surgery	47.25 ± 25.87		62.31 ± 24.48		65.29 ± 22.89		68.17 ± 19.23		79.69 ± 17.12		82.25 ± 14.98	
Adjusted mean difference [95% CI] (p-value)	0.99 [− 6.19, 8.17] (0.79)											
**SF-36 (BP)**												
Surgery	20.44 ± 17.15		58.77 ± 22.70		68.47 ± 19.69		73.48 ± 22.37		71.79 ± 20.34		73.07 ± 22.21	< .001
Non-surgery	33.52 ± 16.70		53.08 ± 24.21		61.76 ± 23.25		67.83 ± 19.95		75.73 ± 18.23		76.13 ± 17.19	
Adjusted mean difference [95% CI] (p-value)	3.13 [− 3.45, 9.71] (0.35)											
**SF-36 (GH)**												
Surgery	53.07 ± 18.99		61.23 ± 19.19		62.86 ± 20.34		65.37 ± 19.86		58.59 ± 19.37		66.70 ± 17.85	0.04
Non-surgery	50.92 ± 17.47		55.31 ± 17.54		51.03 ± 17.31		58.17 ± 20.40		62.29 ± 18.04		66.08 ± 17.13	< .001
Adjusted mean difference [99% CI] (p-value)			3.93 [− 3.96, 11.82] (0.99)		8.01 [− 0.73, 16.74] (0.09)		1.15 [− 8.07, 10.38] (1.00)		− 7.08 [− 15.52, 1.35] (0.15)		− 3.01 [− 11.4, 5.38] (1.00)	
**SF-36 (RP)**												
Surgery	15.35 ± 27.85		39.15 ± 39.38		51.53 ± 40.64		64.02 ± 37.10		60.33 ± 41.69		17.05 ± 28.41	< .001
Non-surgery	22.89 ± 32.39		39.23 ± 40.26		45.59 ± 41.96		72.50 ± 36.17		59.90 ± 41.17		27.45 ± 35.45	
Adjusted mean difference [95% CI] (p-value)	2.75 [− 5.56, 11.07] (0.52)											
**SF-36 (RE)**												
Surgery	38.01 ± 36.43		59.12 ± 44.16		67.35 ± 41.66		73.98 ± 41.17		59.42 ± 48.13		6.06 ± 21.89	< .001
Non-surgery	48.36 ± 40.15		60.51 ± 42.45		61.76 ± 43.53		86.67 ± 28.50		67.36 ± 45.35		22.22 ± 40.37	
Adjusted mean difference [95% CI] (p-value)	− 0.80 [− 10.35, 8.75] (0.87)											
**SF-36 (SF)**												
Surgery	35.75 ± 20.24		58.02 ± 22.61		69.39 ± 21.51		75.61 ± 22.18		74.73 ± 23.35		79.83 ± 22.59	< .001
Non-surgery	47.18 ± 22.97		58.08 ± 23.53		60.29 ± 22.92		70.83 ± 18.08		79.69 ± 20.08		76.47 ± 19.63	
Adjusted mean difference [95% CI] (p-value)	2.65 [− 3.89, 9.20] (0.43)											
**SF-36 (MH)**												
Surgery	49.54 ± 18.19	67.77 ± 17.67		71.84 ± 17.28		75.41 ± 17.74		73.13 ± 17.32		76.18 ± 16.32		< .001
Non-surgery	56.85 ± 18.13	64.68 ± 15.63		64.59 ± 17.54		67.87 ± 15.84		74.58 ± 12.03		73.88 ± 14.56		
Adjusted mean difference [95% CI] (p-value)	3.96 [− 0.58, 8.49] (0.09)											

*VAS-B* visual analogue scale for back pain, *VAS-L* visual analogue scale for leg pain, *K-ODI* Korean version of the Oswestry Disability Index, *EQ-5D score* EuroQol 5-Dimension instrument score, *EQ-VAS* EuroQol visual analogue scale, *SF-36* 36-Item Short-Form Health Survey.

The results from the linear mixed effects model (*adjusted mean difference between surgery and non-surgery cohort, confidence intervals are represented according to statistical significance; †cohort difference; ‡ time difference).

^††^The SF-36 consists of eight sections, including vitality (VT), physical functioning (PF), bodily pain (BP), general health perceptions (GH), physical role functioning (RP), emotional role functioning (RE), social role functioning (SF) and mental health (MH).

### Characteristics of the surgery cohort

To identify distinctive characteristics of the surgery cohort, demographic factors and baseline PROs were compared between cohorts. Age (hazard ratio [HR] 0.97 [95% CI 0.95–0.99]; p < 0.01) and SF-36 (PF) scores (HR for 5-point change 0.89 [95% CI 0.86–0.93]; p < 0.01) were factors that showed significant relationships with the surgery cohort. Based on the analysis, the following equation was derived.

$$Surgery preference score =age\times \left(-0.02988\right)+SF\_36(PF)\times (-0.023)$$

According to the surgery preference score, the optimal cutoff for discriminating the surgery cohort from the nonsurgery cohort was − 2.3. The probability of surgery within 3 months in patients with a surgery preference score > − 2.3 was 67% (95% CI 55–80%) (p < 0.01).

### Complications

No violation of the study protocol was reported by the researchers or the DSMB board. One participant in the surgery cohort complained of neuropathic pain for 1 month after surgery, which was controlled with medication and epidural injection. No participants experienced surgical complications such as wrong level surgery, dura tear, infection, hematoma, or neurological injury.

## Discussion

The present study was designed as a comprehensive cohort study for comparing outcomes between nonsurgical treatment and surgery in surgical candidates in a prospective manner while respecting patients’ treatment preferences. All outcomes significantly improved after either surgery or nonsurgical treatment over 24 months, but surgery resulted in faster improvement regarding VAS-B and VAS-L. A dramatic effect of surgery on VAS-B and VAS-L was achieved within 1 month after surgery, but the effect leveled off thereafter. The outcomes between nonsurgery and surgery patients became similar during 24 months of follow-up. Because the design of the CCS respected patients’ preferences, and the preference may be characterized by comparing the surgery and nonsurgery cohorts. A surgery preference score of more than -2.3 had a positive predictive value of 67%, and modification of the preference score may be used as a supporting tool in the decision-making process regarding whether to perform surgery^32^.

### Nonsurgical treatment and outcomes

The Maine Lumbar Spine Study showed that surgery yielded better improvements in the predominant symptom (71% vs. 43%, p < 0.01) at 1 year and (70% vs. 56%, p < 0.01) at 5 years postoperatively^8,9,33^. Patients were more satisfied with their current status at both 5 years (63% vs. 46%, p < 0.01) and 10 years (71% vs. 56%, p < 0.01) postoperatively^8,9^. The SPORT trial also showed that the satisfaction rates of participants were higher after surgery than after nonsurgical treatment at 3 months (68% vs. 29%), 1 year (71% vs 44.7%), and 2 years (72% vs. 49%)^6^. Lurie et al. reported that the treatment effect of surgery was seen as early as 6 weeks, appeared to reach its maximum by 6 months, and persisted over 8 years in an as-treated analysis after both surgical and nonsurgical treatment, but the effect was better after surgery over 8 years^7^. These prospective studies support better outcomes of surgery than nonsurgery during short to medium follow-up periods. However, the Maine Lumbar Spine Study showed similar improvement after either surgery or nonsurgery (69% vs. 61%, p = 0.2) at 10 years postoperatively^8,9,33^. According to a systematic review by Jacobs et al., surgery led to faster pain relief, but there was no difference in outcomes at 1 and 2 years after either surgery or nonsurgical treatment^10^. In summary, surgery ameliorated symptoms faster than nonsurgical treatment, but the long-term outcomes were similar between nonsurgical and surgical management^10,34–36^.

In the clinic, physicians encountered requests for nonsurgical treatment from surgical candidates. However, we were not sure about whether to recommend nonsurgical management in this clinical setting^6–9,33^. The present study was designed to address this issue in a specific setting. As expected, the present study also showed that nonsurgical management could significantly improve symptoms even in surgical candidates. Although surgery provided faster and better improvement of back and leg pain than nonsurgical treatment at 1 month, the effect of both treatments became the same thereafter for 24 months. Similar results of the present study also supported a limited role of surgery for LDH. However, we noted that only half of the surgical candidates actually underwent surgery, which underscores the importance of revising the indications for surgery. The apparent lack of difference between the nonsurgery and surgery cohorts following better initial improvements in surgery cohort than in nonsurgery cohort might not show a maximum effect of surgery in a short term but might reveal a “floor effect” of the current measurement instruments^7,37,38^. Although only half of the surgical candidates opted to undergo surgery, the current surgical indications could not be revised based on the results from current measurement instruments. In this regard, we discussed the “floor effect” of the instruments, but this effect was not verified or discussed elsewhere^37,38^.

### Preferences for surgery

It is generally accepted that surgery is necessary for 10% of patients with LDH, and natural improvement could be expected in the others^11,12^. This information may have led surgical candidates to opt for nonsurgical management. However, this rate of 10% applies to all cases of LDH, regardless of severity, and the rate among surgical candidates may be higher^6,40,42^. In the SPORT trial, when surgical candidates were randomly allocated to the surgery or nonsurgery cohort, 60.3% (140/232) in the surgery cohort and 55.4% (133/240) in the nonsurgery cohort followed their allocation^11^. When we regard random allocation of surgical candidates as the surgeons’ decision, we may assume that approximately one-half of patients would have a different opinion from physicians/surgeons^11^. When surgical candidates were free to choose their treatment, 26.6% (191/719) of participants ultimately received nonsurgical treatment in the SPORT trial^6^. The present study also showed that 55% of surgical candidates selected nonsurgical treatment. Therefore, the proportion of patients who opt for surgery may be higher than 10% among surgical candidates. The next issue is how to identify the preferences of surgical candidates. Classical symptoms and imaging findings are informative but are not sufficient as decision-support tools^34,43^. The present cohorts may be regarded as representing participants’ preferences. We hypothesized that contrasting features between the cohorts may provide clues as to how to measure patient preference. Age and SF-36 (physical function) scores reflected these preferences, with a Harrell c-index of 67% (95% CI 0.61–0.76). Interestingly, the pain score was not related to patient preference. The specific factors may not be applicable for every spine clinic, but the concept of using PRO-based parameters may be applicable in assessing patient preferences.

### Strengths and limitations

#### Strengths

The present study may be meaningful in presenting results from a specific setting; the patients were surgical candidates who were recommended to undergo surgery by spine physicians but requested a second opinion. Since surgery is the normal course of treatment under current guidelines, the intervention addressed in the present study was not surgery but a nonsurgical treatment. Although the results are similar to those of previous studies, the results obtained from this specific setting may be helpful in discussing nonsurgical options with surgical candidates. In addition, the design of the CCS enabled us to assess the preferences for surgery among surgical candidates.

### Limitations

First, this study was an interim analysis at a 1-year follow-up. The small size of the study cohort and the nonrandomized cohorts made the study subject to type I or II error. We are preparing results from randomized cohorts with a longer follow-up period than the present one. However, a high drop-out rate may bias the results. The present study did not provide any reward to the patients, and their access to the clinic was the same as that of regular patients. Although research nurses tried to contact participants to obtain clinical information and remind them about appointments, not all participants visited the clinic on a regular basis or responded to telephone contact. The follow-up rate might have been improved by providing rewards or managing participants’ clinic visits separately, but those measures might also have disrupted the natural flow of patient care and were not considered for the present study. Second, the level of sports activities among the participants could have influenced the outcomes but was not assessed in this study. Although sports activities were not assessed, they were limited in all patients until pain became tolerable, and the influence of sports activities might not be significant. Third, the present results were not very different from previous results. We specified surgical candidates for inclusion in the present study to specify the role of surgery, but the outcomes were not new. Although we discussed the floor effect of the current tools, the hypothesis has not been verified or discussed elsewhere^37,38^. Nonetheless, we suggest that subsequent studies may consider using different instruments so as to show an outcomes in a different perspective^6,11,32,39–41^. Fourth, although we attempted to determine patient preference based on PROs, PROs are not primarily intended to reveal psychological factors^41,44^. Moreover, the positive predictive probability of the preference score was 67%, and the factors included may not be generally applicable. Nonetheless, the concept of utilizing PRO-based parameters in addition to pain, imaging findings, and symptom duration to assess patient preference may be helpful in the decision-making process.

## Conclusion

Although nonsurgical treatment resulted in less improvement than surgery in the short term, the improvement was enhanced in the mid- and long term. When preferred by patients, nonsurgical treatment may be a negotiable option for surgical candidates in the shared decision-making process. To better understand patient preferences, utilizing PRO-based parameters may be helpful.

## Supplementary Information

Supplementary Information
