# Supplementary material for: Nonsurgical treatment outcomes for surgical candidates with lumbar disc herniation: a comprehensive cohort study
Source: Sci Rep. 2021 Feb 16;11:3931. doi: 10.1038/s41598-021-83471-y (PMC7887235; doi:10.1038/s41598-021-83471-y)
Supplement: Supplementary file 1 — Supplementary Information [file 41598_2021_83471_MOESM1_ESM.docx]

Non-Surgical Treatment Outcomes for Surgical Candidates with Lumbar Disc Herniation: a comprehensive cohort study

Chi Heon Kim, MD, PhD^1,2*^; Yunhee Choi, PhD^3*^; Chun Kee Chung, MD, PhD^1,2,4^; Ki-Jeong Kim, MD, PhD^1,5^; Dong Ah Shin, MD, PhD^6,7^; Youn-Kwan Park, MD, PhD^8,9^; **Woo-Keun Kwon, MD, PhD**^8,9^**;** Seung Heon Yang, MD^1,2^; Chang Hyun Lee, MD^1,2^; Sung Bae Park, MD, PhD^1,10^; Eun Sang Kim, MD, PhD^11^; Hyunsook Hong, MS^3^; Yongeun Cho, MD, PhD^7,12^

Supplement 1. Patients and design of study

A prospective study entitled “Comparative Effectiveness Study for Surgery vs. Non-Surgery in Patients with Low Back Pain (CES_SNS_LBP)” was approved by the institutional review board and began in August 2016. The CES_SNS_LBP trial was designed to include patients from five teaching university hospitals (with nine attending spine surgeons) in South Korea. The present study intended to compare nonsurgical and surgical outcomes of lumbar disc herniation (LDH) in patients who voluntarily visited a clinic for a second opinion after surgery was recommended by another physician who actively treats spinal disease (spinal physicians). In clinical research, randomized trials are widely accepted as the definitive method for evaluating the efficacy of therapies.[1] However, in real-world clinical research, many patients do not consent to randomization.[1] The Comprehensive Cohort Study (CCS) is designed to recruit all patients fulfilling the clinical eligibility criteria regardless of their consent to randomization. Thus, the CCS included both randomized and observational cohorts of subjects who consented to participate in study but declined to undergo randomizaiton.[1] The enrollment process consisted of three steps (Table 1): screening according to inclusion/exclusion criteria (Table 1), consent for inclusion in the study and selection of inclusion in the randomization or observational cohorts (Fig 1). Attending surgeons and research coordinators (registered nurses) participated in all steps. Participants were allowed to cross over to the other treatment cohort or to withdraw from participation at any time. A web-based system was used across participating hospitals for randomization and data registration. The surgical team and nurses involved in the study were blinded to their selections, and their choices did not influence treatment. Access to a web-based data management system was limited only to authorized research nurses and statisticians. All surgeries were scheduled within 3 months of the initial clinic visit. The follow-up schedules were the same for both study cohorts and regular patients, and their outcomes were evaluated during clinic visits or via telephone at 1, 3, 6, and 12 months after the initiation of treatment and yearly thereafter. The present study did not provide any reward to patients, and their schedule in the clinic was the same as that of regular patients. The research team tried to ensure study subjects’ follow-up rate by managing clinic schedules and contacting the subjects via telephone while respecting their voluntary participation. As such, any possible negative effects of the study were minimized. The independent data safety monitoring board (DSMB) reviewed the study every 6 months. This study was approved by the institutional ethical review board of each university hospital (H 1605-013-759, 4-2106-0492, and B1603/337-004) and registered at both clinicaltrials.gov (NCT02883569, first posted on Aug/30/2016) and the Clinical Research Information Service (https://cris.nih.go.kr/cris/en/) (KCT0000203). All research was performed in accordance with relevant laws/guidelines/regulations of the Republic of Korea, and the present study was conducted in accordance with the principles of the Declaration of Helsinki. Written informed consent was obtained from all participants and/or their legal guardians.

Supplement 2. Statistical analysis

An interim analysis was planned to meet the requirements of governmental funding in the 2^nd^ year and to decide whether to extend the study for a longer follow-up period. Because in the present study the proportions of crossover between the surgery and nonsurgery cohorts were fairly high (67% and 31% from the surgical and nonsurgical treatment cohorts), the outcomes of the actually received treatments were analyzed (as-treated analysis), with comparison between as-treated (actual treatments received) surgery cohort and the non-surgery cohort. The primary endpoint was successful pain relief, which was defined as a decrease in the pain score of more than 2.5 or a final raw pain score of less than 3.5 at a 2-year follow-up. It was calculated that 136 participants in each group were necessary to have a power of 85% with a two-tailed significance level of 0.05, expecting that 85% and 70% of patients receiving surgical and nonsurgical treatment, respectively, would have successful pain relief. The sample size was determined to be 340 considering a drop-out rate of 20%. An intention-to-treat analysis for the randomized group was planned, and the results were compared with the nonrandomized group for external validity. The outcomes of the interim analysis were the changes in VAS-B, VAS-L, K-ODI, EQ-5D, EQ-VAS, and each section of SF-36 from the baseline measures during the follow-up period. The number of patients with available data in each follow-up period was not the same because not all patients visited the clinic at all planned appointments, and they could not always be reached via telephone when they missed a clinical appointment. Therefore, a generalized linear mixed-effect model was utilized to compare clinical outcomes between the surgery and nonsurgery cohorts and to address a patient-specific trend in the outcomes. The fixed effects were the cohort (surgery vs. nonsurgery), measurement time, the interaction between the cohort and measurement time, and confounding variables such as age, sex, BMI, smoking status, diabetes, the centers participating in this study, and the baseline measurement corresponding to the outcome for each linear mixed model. The random effect was a participant. Adjusting the cofounding variables, the adjusted mean difference between the surgery and nonsurgery cohorts was estimated based on the mixed models. When group comparisons were performed at each measurement time due to significant interaction between the cohort and measurement time, the adjusted p-value and 99% confidence interval were estimated by the Bonferroni method to control type I error inflation due to multiple testing. Marginal Cox regression analysis, accounting for the cluster effect caused by the study centers, was performed to identify significant factors affecting the time from enrollment to surgery and to create a formula for the surgery preference score.[2] Demographic factors and baseline clinical parameters were considered, and factors with a p-value of < 0.1 were included in the multivariable analysis. The final model was decided using a stepwise method. The characteristics of cohorts were compared using the chi-square test for noncontinuous values and the t-test or Wilcoxon rank-sum test for continuous values. Categorical variables are summarized as frequencies (%). Continuous data were summarized as mean or median (min, max) values depending on their normality. Based on the hazard ratio of significant factors characterizing the surgery cohort, a formula for the surgery preference score was produced. The optimal cutoff value of the surgery preference score to discriminate the surgery cohort from the nonsurgery cohort was determined using the minimum p-value approach and validated using twofold cross validation.[3,4] All statistical analyses were performed using SAS version 9.4 (SAS Institute, Cary, NC, USA), and statistical significance was defined as p < 0.05 (two-sided).

References

1 Schmoor, C., Olschewski, M. & Schumacher, M. Randomized and non-randomized patients in clinical trials: experiences with comprehensive cohort studies. *Stat Med*. **15**, 263-271, (1996).

2 Lee, E. W., Wei, L. J., Amato, D. A. & Leurgans, S. *Cox-Type Regression Analysis for Large Numbers of Small Groups of Correlated Failure Time Observations*. 237-242 (Kluwer Academic Publishers, 1992).

3 Contal, C. & O'Quigley, J. An application of change point methods in studying the effect of age on survival in breast cancer. *Comput Stat Data Anal*. **30**, 253-270, (1999).

4 Faraggi, D. & Simon, R. A simulation study of cross-validation for selecting an optimal cut-point in univariate survival analysis. *Stat Med*. **15**, 2203-2213, (1996).
